# Real-world evidence for improved outcomes with histamine antagonists and aspirin in 22,560 COVID-19 patients

**DOI:** 10.1038/s41392-021-00689-y

**Published:** 2021-07-14

**Authors:** Cameron Mura, Saskia Preissner, Susanne Nahles, Max Heiland, Philip E. Bourne, Robert Preissner

**Affiliations:** 1 School of Data Science and Department of Biomedical Engineering, University of Virginia, Charlottesville, VA USA; 2 Department Oral and Maxillofacial Surgery, Charité–Universitätsmedizin Berlin, corporate member of Freie Universität Berlin, Humboldt-Universität zu Berlin, and Berlin Institute of Health, Berlin, Germany; 3 Institute of Physiology and Science-IT, Charité–Universitätsmedizin Berlin, corporate member of Freie Universität Berlin, Humboldt-Universität zu Berlin, and Berlin Institute of Health, Berlin, Germany

**Keywords:** Predictive medicine, Gastroenterology, Infectious diseases, Drug discovery

**Dear Editor**,

The COVID-19 pandemic has driven great interest in the therapeutic potential of repurposed drugs with well-established benefits and safety profiles (toxicity, bioavailability, etc.), many of which act via signal transduction pathways. One category of such drugs is those that reduce acid production in gastroenterological contexts. Acid-suppressing drugs belong to two main classes, based on their mechanisms of action: (i) proton-pump inhibitors (PPIs) sterically block H^+^/K^+^-ATPase pumps, impeding the final step of acid release in the gastric mucosa. (ii) Histamine H_2_ receptor antagonists (H2RA) competitively bind the H2R, a type of G-protein coupled receptor (GPCR),^1^ and block the natural stimulation of its downstream signal transduction cascade by histamine; famotidine (e.g., Pepcid^®^) and ranitidine (e.g., Zantac^®^) are exemplary H2RAs.

A dense web of functional linkages exists between histamine and H2RAs, on the one hand, and disparate physiological pathways on the other hand. These downstream signaling pathways include gastrointestinal systems (acid reduction) as well as the dysregulated inflammatory cascades (cytokine storm) that likely underlie much of the pathophysiology of COVID-19.^1^ The mechanistic basis of a putative role of famotidine in COVID-19 likely involves its roles as an H2RA versus, for instance, direct binding to the viral protease 3CL^pro^ (and resultant inhibition), as had been originally suspected from molecular docking studies.

Given its many possible mechanistic and regulatory linkages to signal transduction pathways, is famotidine beneficial in treating COVID-19, as gauged by outcomes involving either (i) *infection transmissibility*, (ii) *disease severity indicators* (e.g., likelihood of cases reaching the point of ventilation, WHO severity index), or (iii) *mortality rates*? This question remains unresolved, though not for lack of effort: since a pioneering report^2^ of positive clinical outcomes with famotidine use in COVID-19, over 10 studies have considered the potential therapeutic benefits of famotidine. As we recently reviewed,^3^ many of these reports concluded in favor of famotidine use, others found little to no association between famotidine (or PPIs) and 30-day mortality, and a recent study found a negative association for both PPIs and famotidine. These independent studies have been retrospective and observational; most were cohort-based, with some as case-series (e.g., symptom tracking across longitudinal data); most evaluated inpatient cases; and most attempted to account for confounders and other biases (e.g., via propensity-score matching). Given the conflicting reports thus far, particularly the evidence suggesting a beneficial impact of famotidine on mortality and overall disease progression (e.g., mechanical ventilation), we have undertaken the new analysis reported herein.

Note that all three parallel tracks of findings—those indicating for and against famotidine, as well as neutral (i.e., no association)—rest upon substantially smaller datasets than were drawn upon in the present work. Are any beneficial effects of famotidine detectable on population-wide, international scales? Is it synergistic to treat with famotidine in conjunction with aspirin, a general-purpose anti-inflammatory? Does famotidine use correlate with any measurable parameters that may serve as biomarkers, perhaps offering mechanistic clues (e.g., serum C-reactive protein [CRP] levels as a proxy for inflammation and the cytokine storm)? This work seeks to address these questions.

We began by retrieving data from the COVID-19 Research Network supplied by TriNetX, comprising ≈400 M patients across 30 countries. This federated health research network supplies electronic medical records (diagnoses, procedures, medications, etc.) as aggregated counts of de-identified information. We analyzed a cohort of 22,560 COVID-19 patients taking H_1_/H_2_ receptor antagonists, with a special focus on 1,379 severe cases requiring respiratory support (see CONSORT flow diagram, Supplementary Fig. 1). We defined ‘death’ as the primary outcome, and sought to mitigate confounder bias via propensity-score matching to achieve stratified and balanced sub-cohorts across age and gender (see Supplementary Methods). A total of *n* = 257,864 COVID-19 cases were considered; of these, (i) 7,479 died, (ii) 18,624 used famotidine, (iii) 8,335 used cetirizine, (iv) 3,928 used loratadine, (v) 23,148 used aspirin, and (vi) 5,955 used aspirin and famotidine. Measures of association, risk ratios (RRs) and odds ratios (ORs), along with their respective 95% confidence intervals (CIs), were calculated, as were Kaplan–Meier survival curves.

We statistically analyzed outcomes for treatment with (i) the H1RAs loratadine (e.g., Claritin^®^) and cetirizine (e.g., Zyrtec^®^), (ii) the H2RA famotidine, (iii) aspirin, and (iv) a combination of famotidine and aspirin, as shown in Table 1. For cases that reached the point of respiratory support, we found a significantly reduced fatality risk for famotidine treatment (OR 0.73, CI 0.57–0.94; Table 1, Supplementary Files 1–4). Dual-histamine receptor blockade, concurrently targeting the H_1_ and H_2_ receptors, has been thought to improve COVID-19 clinical outcomes^4^; however, significant improvements were not seen in our cohorts, versus famotidine alone (OR 0.75, CI 0.39–1.46; Supplementary Files 5–8). Notably, and perhaps unexpectedly, the combination of famotidine and aspirin (344 severe cases before matching) *did* exhibit a significant synergistic survival benefit (OR 0.55, CI 0.39–0.78; Fig. 1; Supplementary Files 9–12). The RR for death decreased by 32.5%—an immense benefit, given the more than 3.8 million COVID-19–related deaths thus far. Note that, because of methodological reasons related to data availability and reduced statistical power upon further stratification, we did not further group cases into sub-cohorts based on disease severity; thus, a limitation of our work stems from the distribution of such severities almost certainly being related to the efficacy of any therapeutic intervention.

**Table 1:** Statistical outcomes for patients requiring respiratory support, considering use/disuse of (i) H_1_- or H_2_-receptor antagonists or aspirin, as well as (ii) a combination treatment with famotidine and aspirin

Drug compound [H_1_ or H_2_ antagonist]	Number of patients in cohort (after matching)	Outcome: Death	Odds ratio (OR)	Confidence interval (CI 95%)	Hazard ratio (HR)
Loratadine [H_1_]	88	29	1.00	0.55–1.87	0.84
Cetirizine [H_1_]	95	25	0.85	0.45–1.61	0.80
Famotidine [H_2_]	563	161	0.73	0.57–0.94	0.75
Aspirin (Asp)	527	165	0.79	0.61–1.02	0.71
Famotidine + Asp	305	83	0.55	0.39–0.78	0.53

Can our findings be reconciled with recent studies of famotidine in COVID-19? A case-series of 10 non-hospitalized patients found that self-administration of famotidine had uniformly beneficial impact on disease trajectories, based on quantitative symptom tracking across longitudinal data.^3^ Retrospective, single-center studies also found promising results, e.g. reduced risk of clinical deterioration (intubation and death) for famotidine usage in 83 and 84 hospitalized COVID-19 patients, corresponding to 9.5 and 5.1% of the analyzed cohorts, respectively. Notably, these past studies^3^ found lower levels of serum markers for severe disease (e.g., ferritin, CRP [see Supp Table 1], procalcitonin) in famotidine groups, consistent with our findings and with a potential role for this H2RA in attenuating cytokine release. Finally, a new systematic review and analysis (of published reports) suggests that famotidine may be beneficial, while two other recent meta-analyses are either neutral or (statistically) inconclusive.^3^

If indeed famotidine is beneficial in a significant share of COVID-19 cases, we suspect this could be because of the capacity of H2RAs to attenuate the pro-inflammatory pathways that become dysregulated upon infection (cytokine storms activate pro-fibrotic pathways; lung damage eventually results). Thus, a role for famotidine in COVID-19 may stem from cellular mechanisms and signaling pathways quite unrelated to its classic therapeutic role in gastroenterology—that, in turn, is an important lesson as regards drug repurposing (from a systems pharmacology perspective), targeted therapeutics, and the general idea of a COVID-19 ‘disease map’.^5^

As SARS-CoV-2 infection rates continue surging worldwide, we desperately need more data on potential therapies. The large, international, multi-center retrospective study reported here, sampling over 250,000 COVID-19 cases, hopefully helps clarify the potential benefit of clinically approved histamine antagonists such as famotidine. We anticipate that at least three prospective, randomized, controlled clinical trials that have been underway (NCT04504240, NCT04370262, and NCT04545008) will illuminate famotidine’s potential therapeutic profile. Given the findings reported here, alongside the cost-effectiveness and mild side-effects of OTC drugs like famotidine and aspirin, we suggest that further prospective clinical trials—perhaps utilizing the aspirin combination reported here (Fig. 1)-—are advisable.

**Fig. 1:**
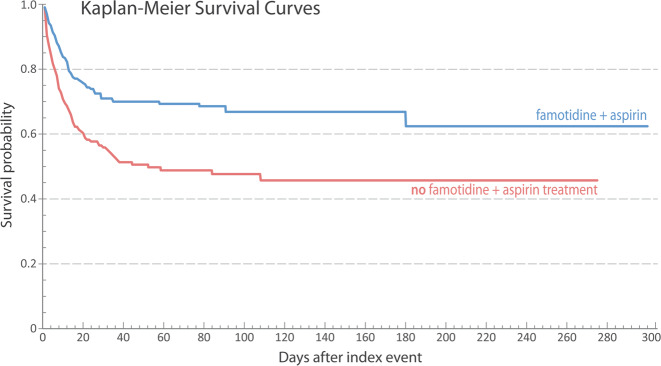
Kaplan–Meier survival curves are shown for COVID-19 patients with (blue) or without (red) the dual combination treatment of famotidine and aspirin; the time-evolution of survival probabilities is given in terms of number of days after the index event (a positive COVID-19 diagnosis)

## Supplementary information

Supplemental Material

Supplemental Files 1-4

Supplemental Files 5-8

Supplemental Files 9-12

## Data Availability

The raw data drawn upon in this work are available from the TriNetX COVID-19 Research Network (https://trinetx.com), comprising ≈400 M patients distributed across 30 countries. This platform supplies electronic medical records (diagnoses, procedures, medications, etc.) as aggregated counts of de-identified information (see Supplementary Methods). In addition, we include 12 Supplementary Data Files that provide data from intermediary stages of analysis in the TriNetX platform, such as various measures of association (famotidine and risk of ventilation, risk differences, RRs and ORs, etc.), cohort statistics, raw data for Kaplan-Meier survival curves, and so on.
