# Supplementary material for: Real-world evidence for improved outcomes with histamine antagonists and aspirin in 22,560 COVID-19 patients
Source: Signal Transduct Target Ther. 2021 Jul 14;6:267. doi: 10.1038/s41392-021-00689-y (PMC8278809; doi:10.1038/s41392-021-00689-y)
Supplement: Supplementary file 1 — Supplemental Material [file 41392_2021_689_MOESM1_ESM.docx]

Supplementary Materials for

Real-world Evidence for Improved Outcomes with Histamine Antagonists and Aspirin in 22,560 COVID-19 Patients

Cameron Mura, Saskia Preissner, Susanne Nahles, Max Heiland, Philip E. Bourne,
Robert Preissner

Correspondence to: [cmura@virginia.edu](mailto:cmura@virginia.edu) or [saskia.preissner@charite.de](mailto:saskia.preissner@charite.de)

**This PDF file includes:**

Materials & Methods

Figure S1

Table S1

**Other Supplementary Materials for this manuscript include the following:**

Data Files S1 – S12 (described on page 4)

**Materials & Methods**

We retrieved data from the COVID-19 Research Network supplied by TriNetX, comprising ≈400M patients from 130 health care organizations in 30 countries. TriNetX offers a global federated health research network that provides access to electronic medical records (diagnoses, procedures, medications, laboratory values, genomic information), and the TriNetX platform uses only aggregated counts and statistical summaries of de-identified information; no protected health information or personal data are made available on the platform. This work was reviewed by our IRB board (UVA IRB tracking ID #23100), who determined that this project did not meet the criteria for Human Subject Research; the IRB deemed no further submission/review necessary to proceed with this project. We analyzed a cohort of 22,560 COVID-19 patients taking H_1_/H_2_ receptor antagonists, with a special focus on 1,379 severe cases requiring respiratory support (see CONSORT flow diagram, Supplementary Figure S1). We defined 'death' as the primary outcome, and, in order to try to mitigate confounder bias, we performed propensity score matching to achieve stratified and balanced sub-cohorts across age and gender; specifically, we balanced cohorts using a nearest-neighbor greedy matching algorithm with a caliper of 0.25 times the standard deviation. A total of *n* = 257,864 COVID-19 cases were considered. Of these, (i) 7,479 died, (ii) 18,624 used famotidine, (iii) 8,335 used cetirizine, (iv) 3,928 used loratadine, (v) 23,148 used aspirin, and (vi) 5,955 used aspirin & famotidine. Measures of association, risk ratios (RRs) and odds ratios (ORs), along with their respective 95% CIs, were calculated. Kaplan-Meier survival curves were also computed for each analysis.

Figure S1.

CONSORT flow diagram for the study.
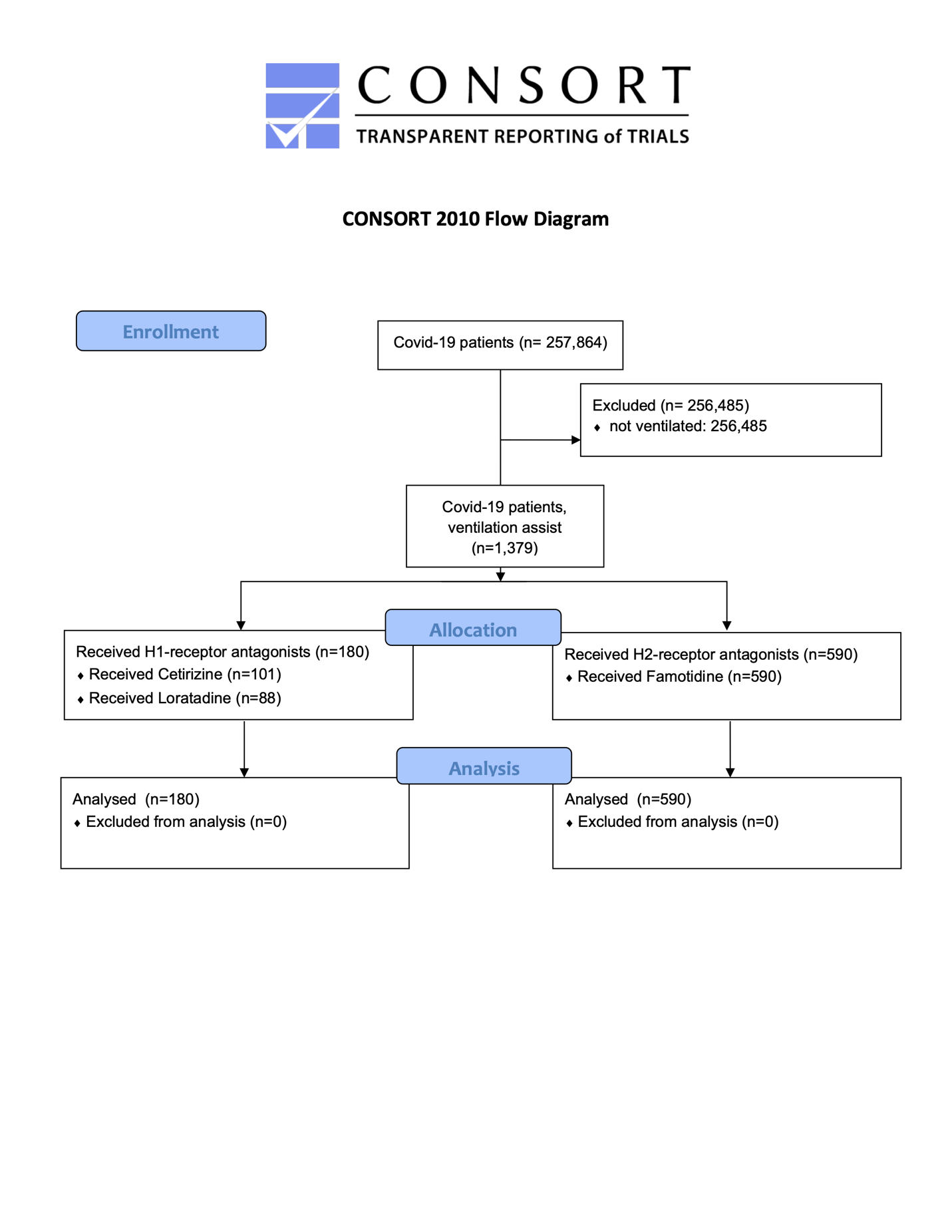


Table S1. Lab values and standard deviations (SD) for serum levels of C-reactive protein (CRP) are given for COVID-19 patients in this study; note that serum CRP levels exceeding ≈10 mg/l are generally indicative of severe infection or disease. Here, we consider six cohorts: Comparing patients who either (1a) died or (1b) survived shows large differences in mean CRP values, as does a comparison of (2a) ventilated versus (2b) non-ventilated patients; perhaps unsurprisingly, those requiring ventilation had CRP levels nearly 2-fold higher than the non-ventilated sub-cohort. Among ventilated patients, the combination of aspirin and famotidine (cohort 3b) is associated with less of a decrease in CRP values (below cohort 3a), versus the differences within other cohort pairs (i.e., cohorts 1a/b and 2a/b); this trend is actually stronger than suggested here, as this cohort is six years older on average and has more comorbidities (data not shown).

| **Cohort** | | **Inclusion criteria** | | **CRP** (mg/l) | **SD** | **Sub-cohort difference** |
| --- | --- | --- | --- | --- | --- | --- |
| COVID-19 | 1a |  | deceased | 100.0 | 107.0 | 65.7 |
|  | 1b |  | survived | 34.3 | 58.0 |  |
| COVID-19 | 2a |  | ✓ ventilation | 74.9 | 98.5 | 35.3 |
|  | 2b |  | ✗ ventilation | 39.6 | 65.6 |  |
| COVID-19 | 3a | ventilation | ✗ aspirin, ✗ famotidine | 81.7 | 104.0 | 13.3 |
|  | 3b | ventilation | ✓ aspirin, ✓ famotidine | 68.4 | 95.4 |  |

Data S1. (The following 12 supplemental items are provided as individual data files, S1–S12.)

- Supplemental File 1: Measures of Association Data Graph for Famotidine
- Supplemental File 2: Measures of Association Data Table for Famotidine
- Supplemental File 3: Kaplan-Meier Raw Data Graph for Famotidine
- Supplemental File 4: Kaplan-Meier Raw Data Table for Famotidine
- Supplemental File 5: Measures of Association Data Graph for H1 and H2
- Supplemental File 6: Measures of Association Data Table for H1 and H2
- Supplemental File 7: Kaplan-Meier Raw Data Graph for H1 and H2
- Supplemental File 8: Kaplan-Meier Raw Data Table for H1 and H2
- Supplemental File 9: Measures of Association Data Graph for Famotidine and Aspirin
- Supplemental File 10: Measures of Association Data Table for Famotidine and Aspirin
- Supplemental File 11: Kaplan-Meier Raw Data Graph for Famotidine and Aspirin
- Supplemental File 12: Kaplan-Meier Raw Data Table for Famotidine and Aspirin
