# Supplementary figures and images for: Real-world evidence for improved outcomes with histamine antagonists and aspirin in 22,560 COVID-19 patients
Source: Signal Transduct Target Ther. 2021 Jul 14;6:267. doi: 10.1038/s41392-021-00689-y (PMC8278809; doi:10.1038/s41392-021-00689-y)

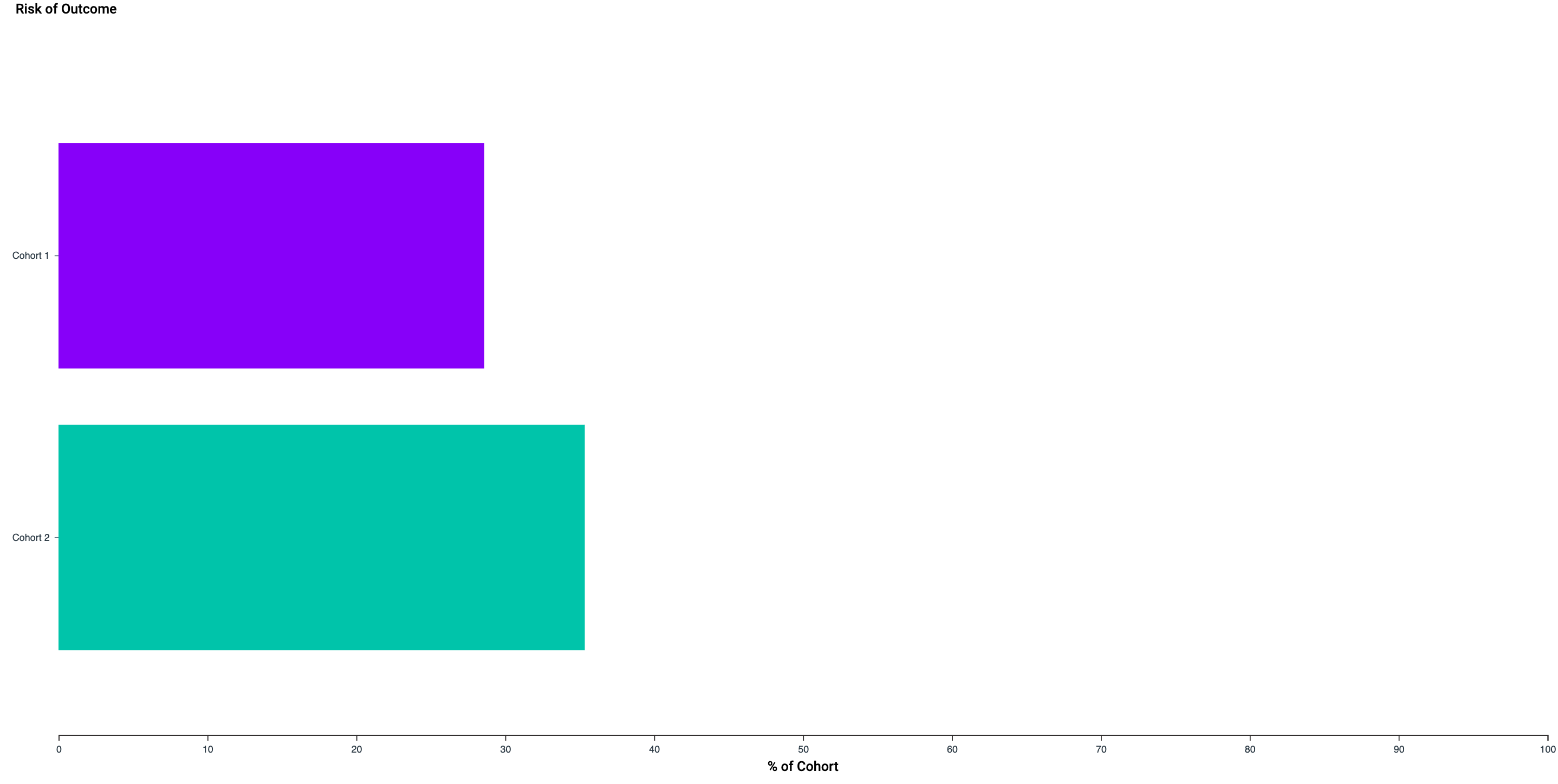

Supplement: Supplementary file 2 — Supplemental Files 1-4 [file 41392_2021_689_MOESM2_ESM.zip › Supplemental-files-famotidine_(vent)/Outcome_1_Result_a_MOA_graph_large.png]

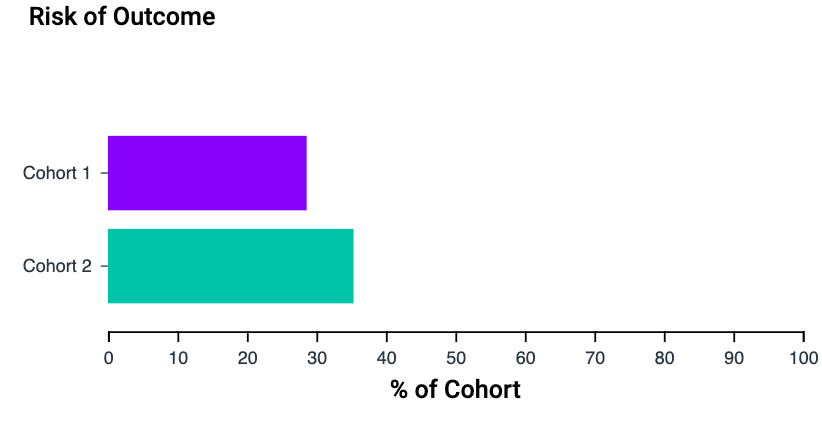

Supplement: Supplementary file 2 — Supplemental Files 1-4 [file 41392_2021_689_MOESM2_ESM.zip › Supplemental-files-famotidine_(vent)/Outcome_1_Result_a_MOA_graph_small.png]

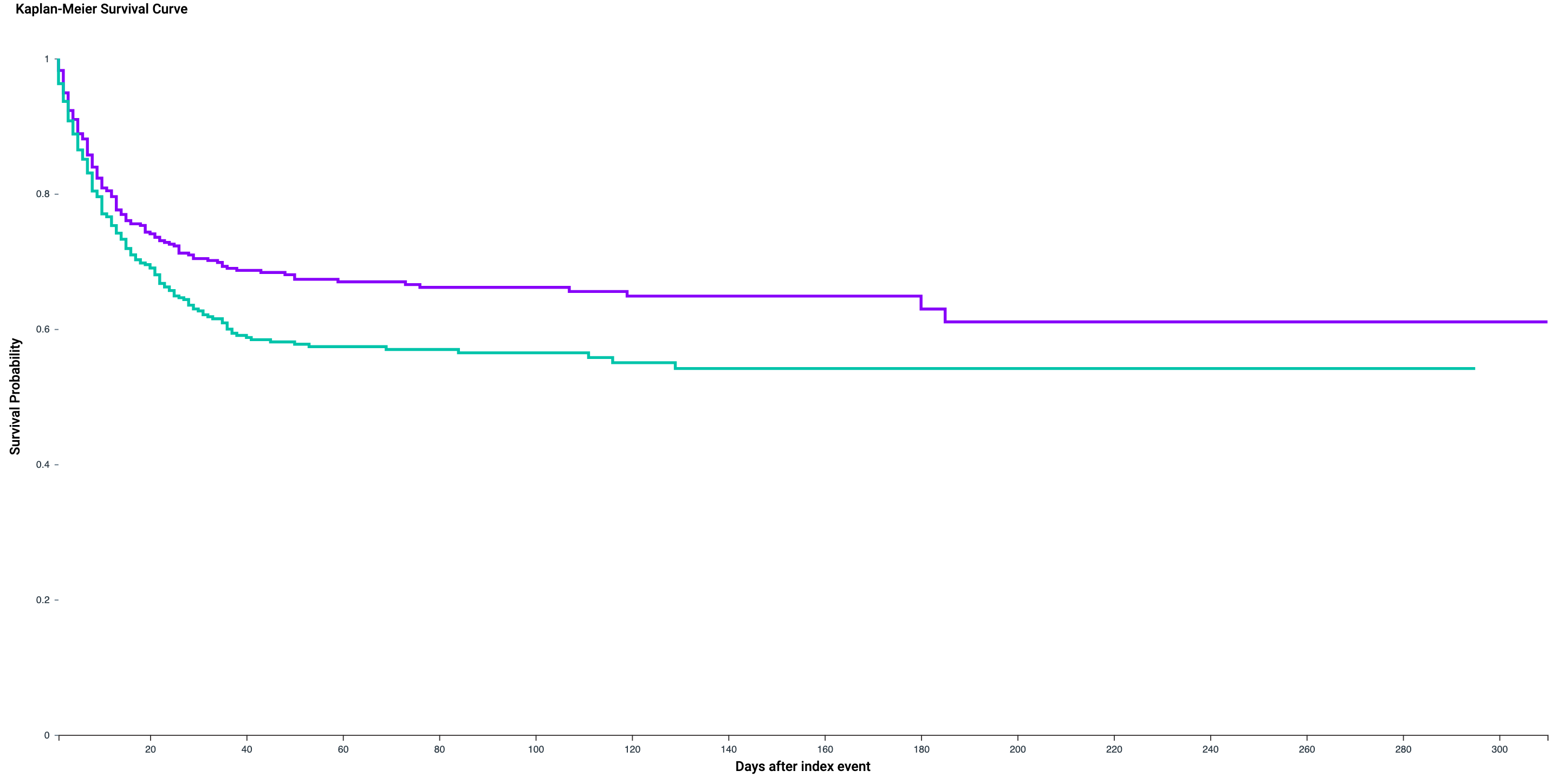

Supplement: Supplementary file 2 — Supplemental Files 1-4 [file 41392_2021_689_MOESM2_ESM.zip › Supplemental-files-famotidine_(vent)/Outcome_1_Result_b_KM_graph_large.png]

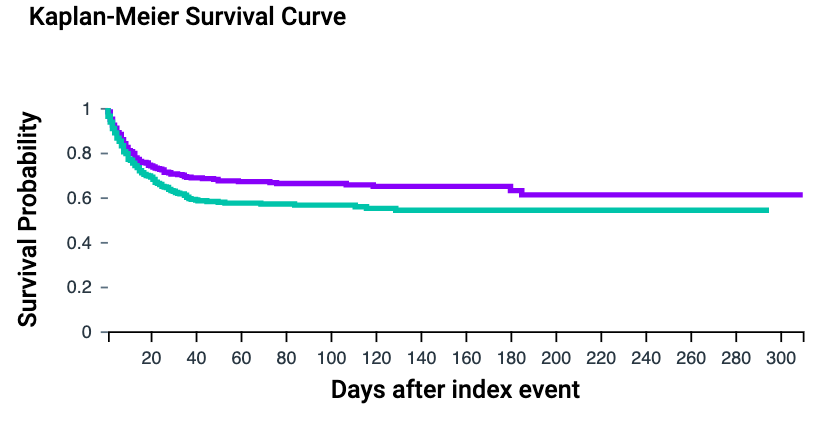

Supplement: Supplementary file 2 — Supplemental Files 1-4 [file 41392_2021_689_MOESM2_ESM.zip › Supplemental-files-famotidine_(vent)/Outcome_1_Result_b_KM_graph_small.png]

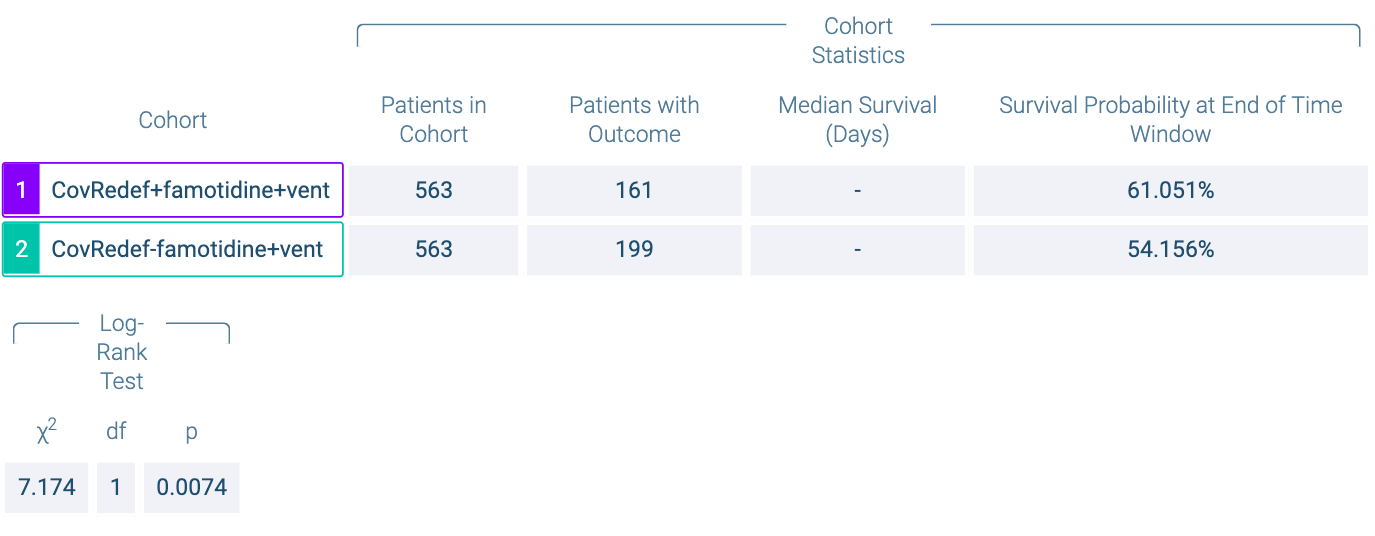

Supplement: Supplementary file 2 — Supplemental Files 1-4 [file 41392_2021_689_MOESM2_ESM.zip › Supplemental-files-famotidine_(vent)/Outcome_1_Result_b_KM_table.png]

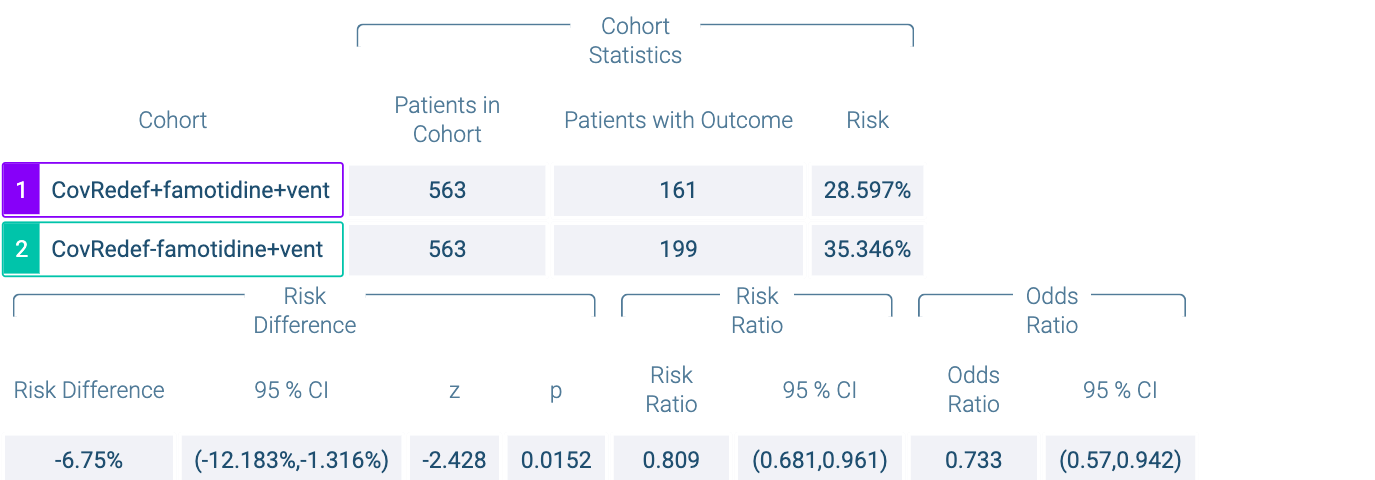

Supplement: Supplementary file 2 — Supplemental Files 1-4 [file 41392_2021_689_MOESM2_ESM.zip › Supplemental-files-famotidine_(vent)/Outcome_1_Result_a_MOA_table.png]

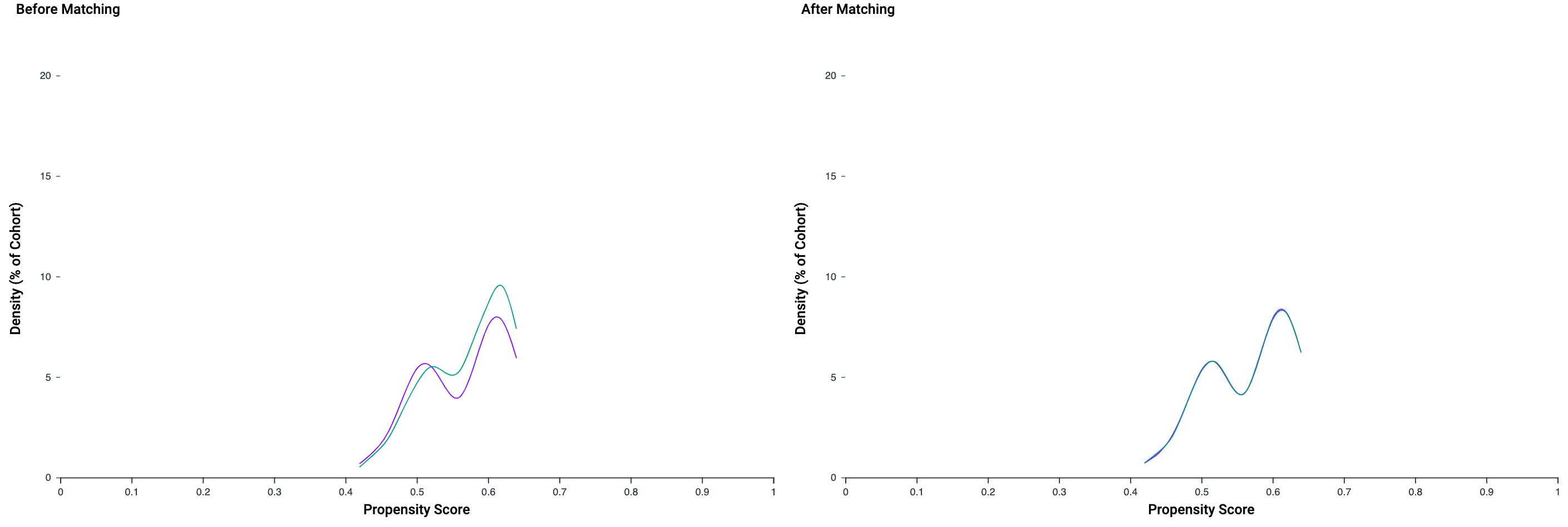

Supplement: Supplementary file 2 — Supplemental Files 1-4 [file 41392_2021_689_MOESM2_ESM.zip › Supplemental-files-famotidine_(vent)/Propensity_Score_Density_Graph_Large.png]

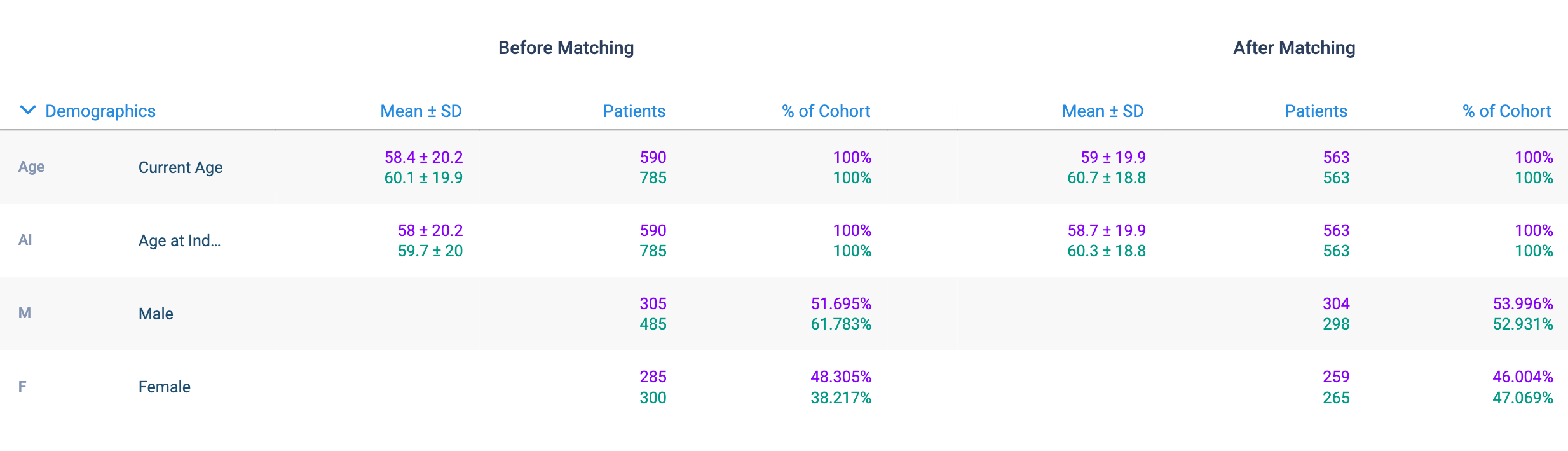

Supplement: Supplementary file 2 — Supplemental Files 1-4 [file 41392_2021_689_MOESM2_ESM.zip › Supplemental-files-famotidine_(vent)/Baseline_Patient_Characteristics.png]

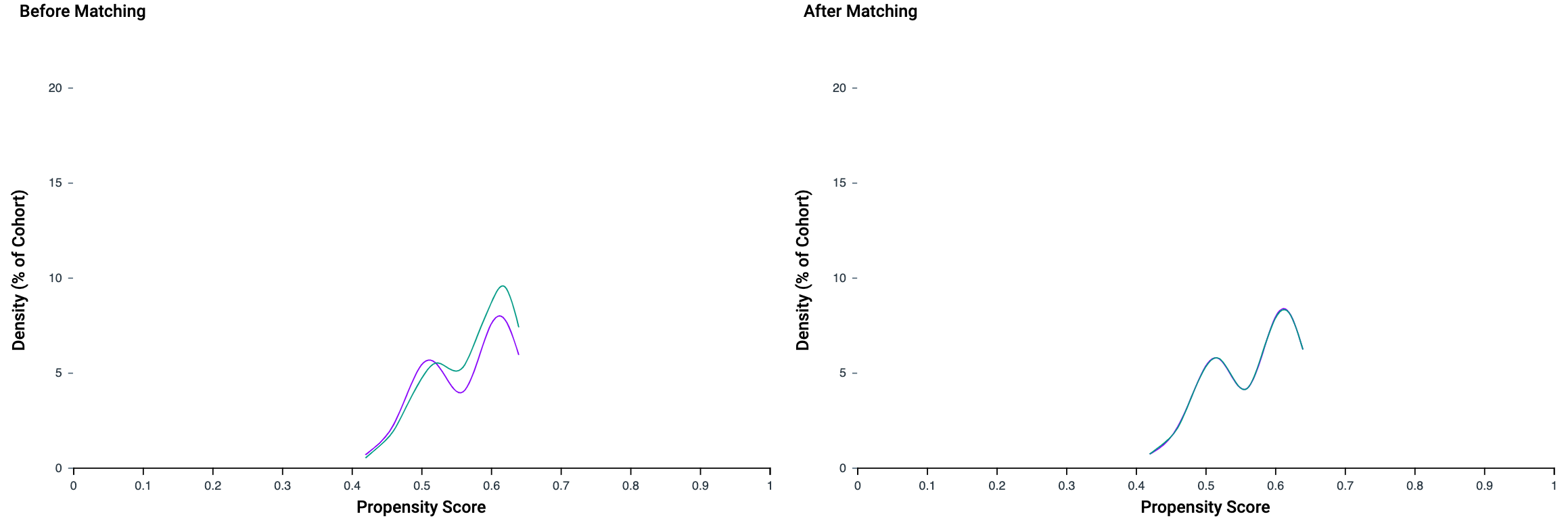

Supplement: Supplementary file 2 — Supplemental Files 1-4 [file 41392_2021_689_MOESM2_ESM.zip › Supplemental-files-famotidine_(vent)/Propensity_Score_Density_Graph_Small.png]

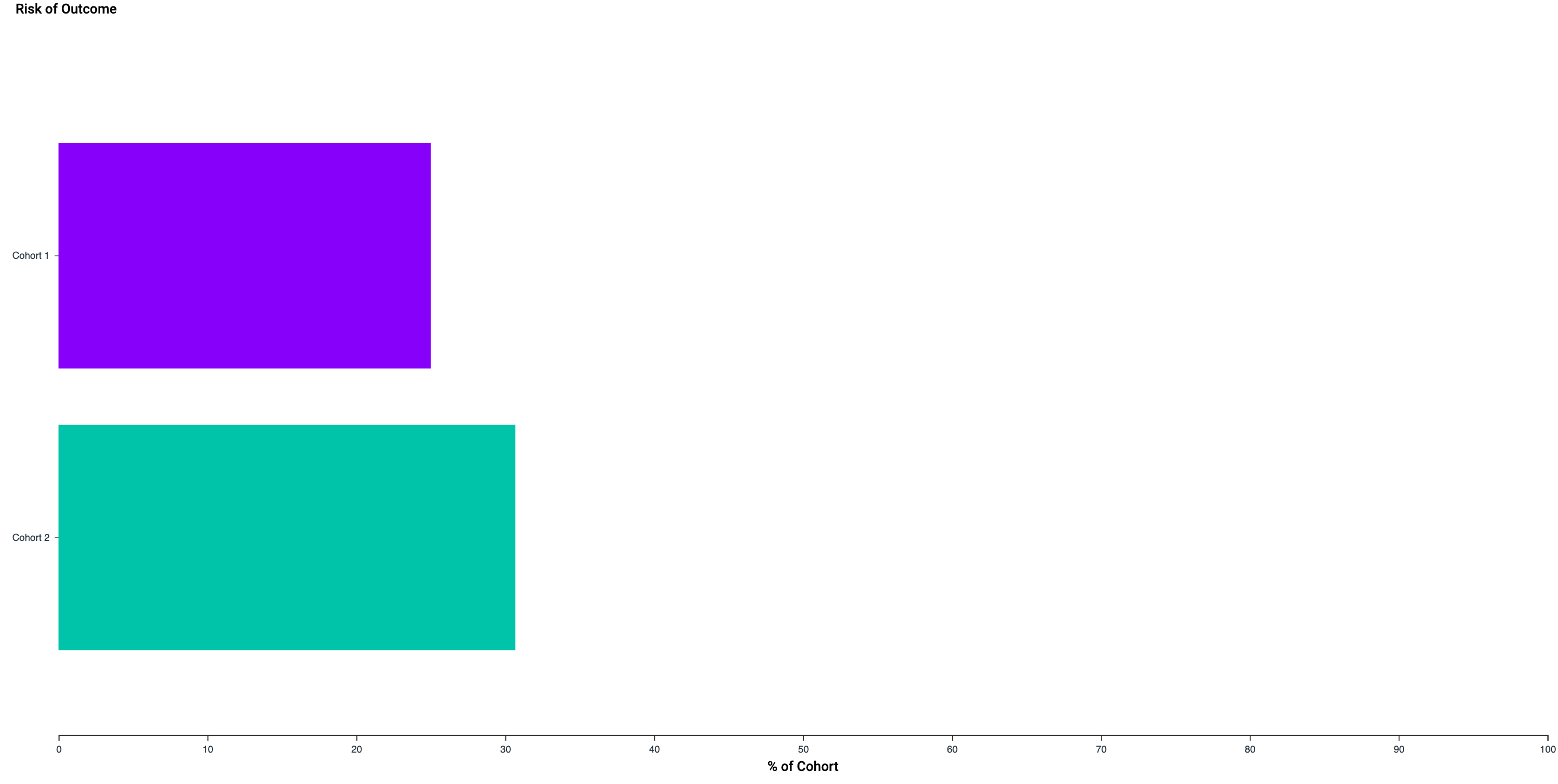

Supplement: Supplementary file 3 — Supplemental Files 5-8 [file 41392_2021_689_MOESM3_ESM.zip › Supplemental-files-H1+H2_(vent)/Outcome_1_Result_a_MOA_graph_large.png]

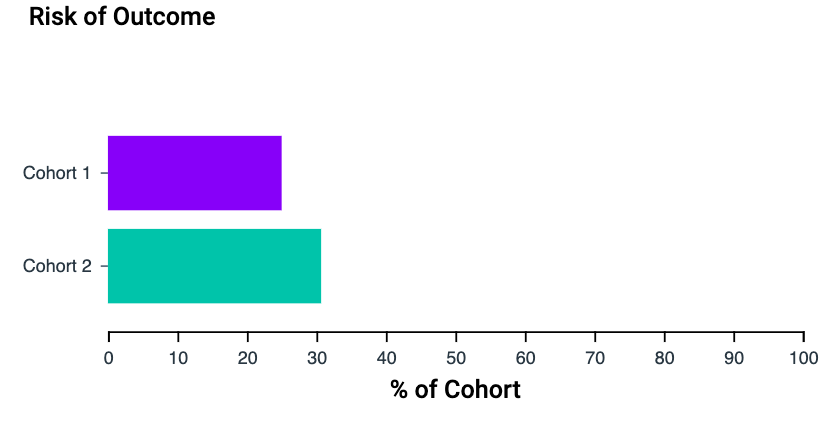

Supplement: Supplementary file 3 — Supplemental Files 5-8 [file 41392_2021_689_MOESM3_ESM.zip › Supplemental-files-H1+H2_(vent)/Outcome_1_Result_a_MOA_graph_small.png]

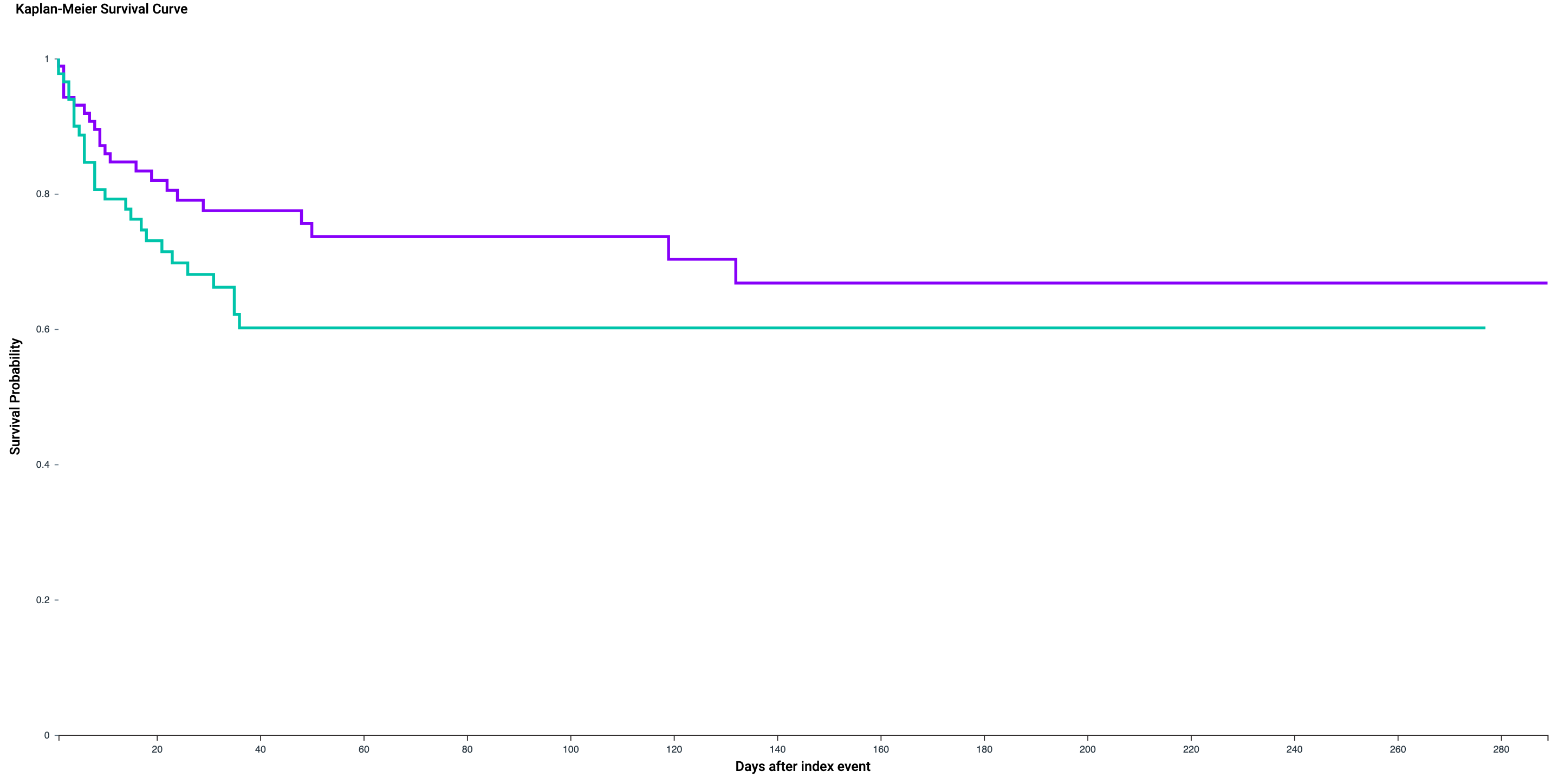

Supplement: Supplementary file 3 — Supplemental Files 5-8 [file 41392_2021_689_MOESM3_ESM.zip › Supplemental-files-H1+H2_(vent)/Outcome_1_Result_b_KM_graph_large.png]

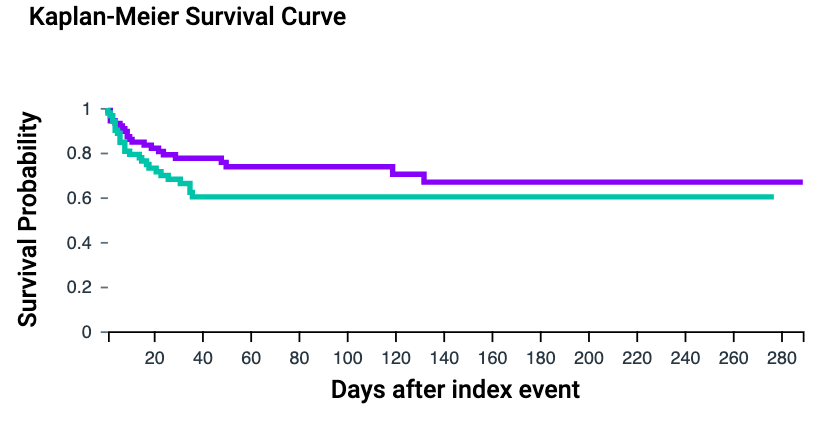

Supplement: Supplementary file 3 — Supplemental Files 5-8 [file 41392_2021_689_MOESM3_ESM.zip › Supplemental-files-H1+H2_(vent)/Outcome_1_Result_b_KM_graph_small.png]

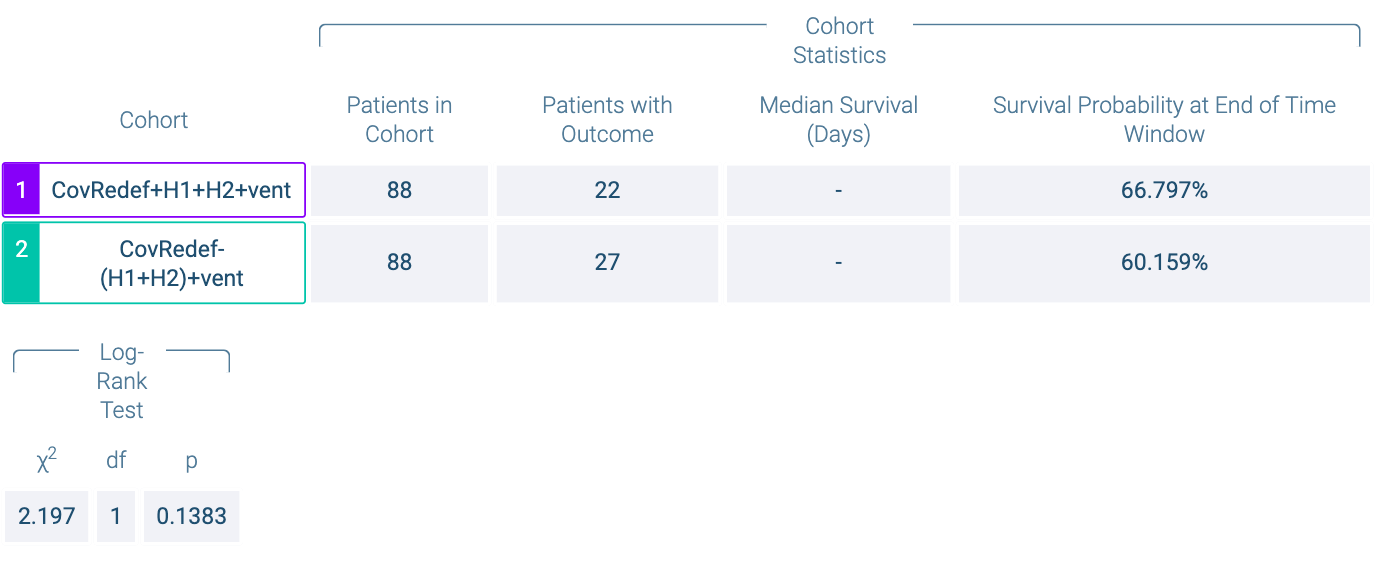

Supplement: Supplementary file 3 — Supplemental Files 5-8 [file 41392_2021_689_MOESM3_ESM.zip › Supplemental-files-H1+H2_(vent)/Outcome_1_Result_b_KM_table.png]

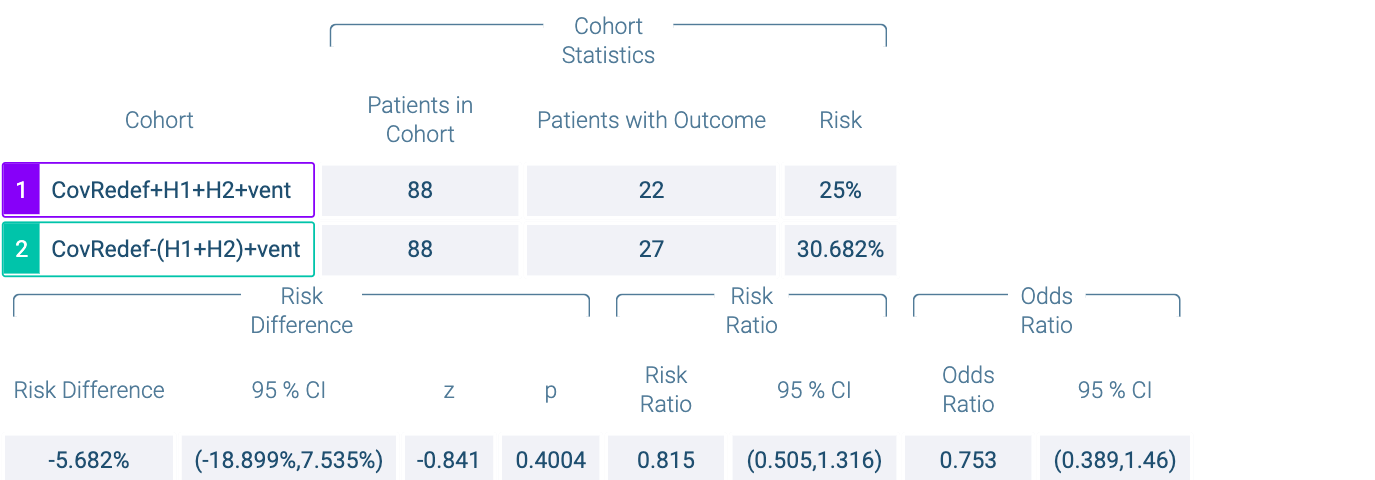

Supplement: Supplementary file 3 — Supplemental Files 5-8 [file 41392_2021_689_MOESM3_ESM.zip › Supplemental-files-H1+H2_(vent)/Outcome_1_Result_a_MOA_table.png]

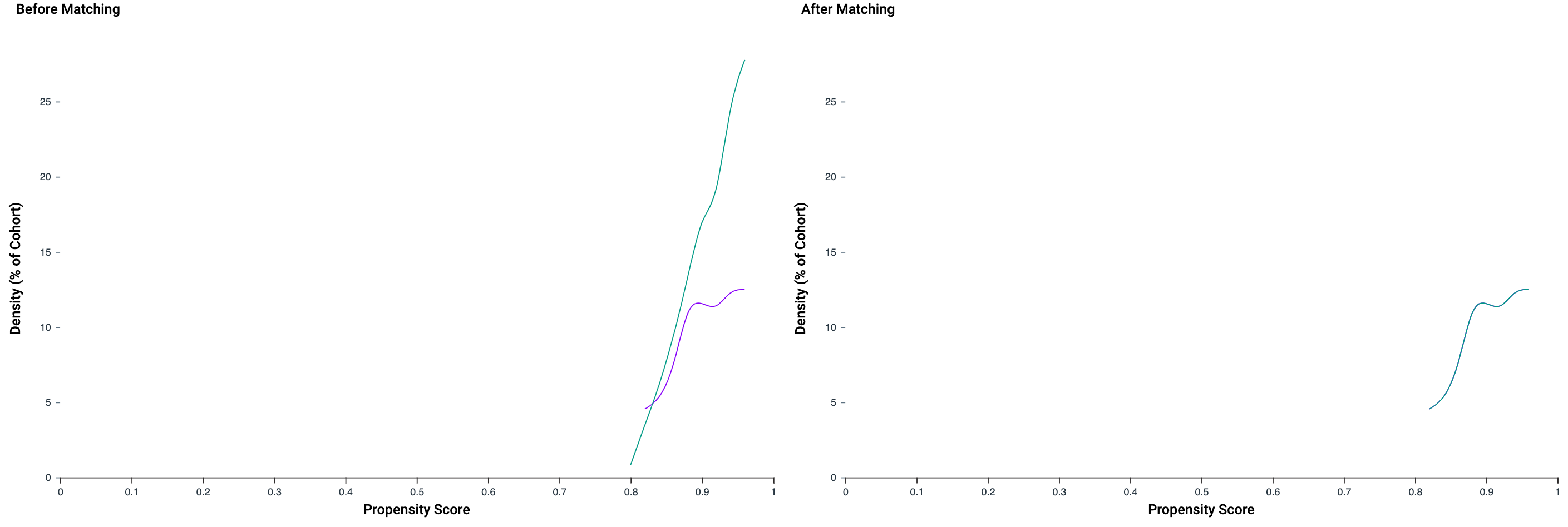

Supplement: Supplementary file 3 — Supplemental Files 5-8 [file 41392_2021_689_MOESM3_ESM.zip › Supplemental-files-H1+H2_(vent)/Propensity_Score_Density_Graph_Large.png]

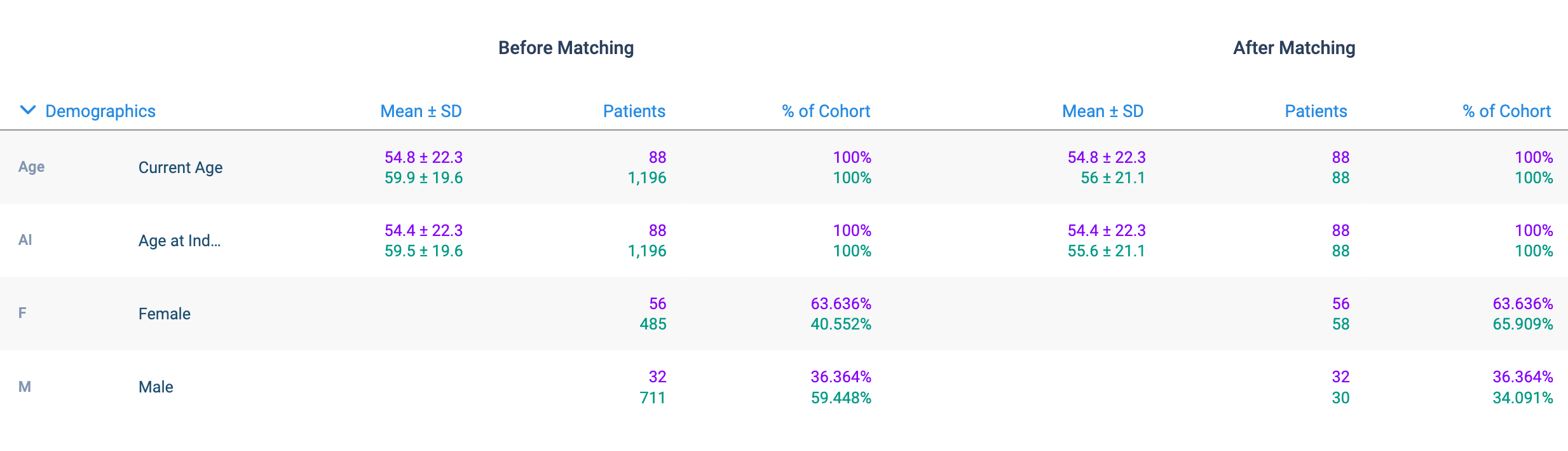

Supplement: Supplementary file 3 — Supplemental Files 5-8 [file 41392_2021_689_MOESM3_ESM.zip › Supplemental-files-H1+H2_(vent)/Baseline_Patient_Characteristics.png]

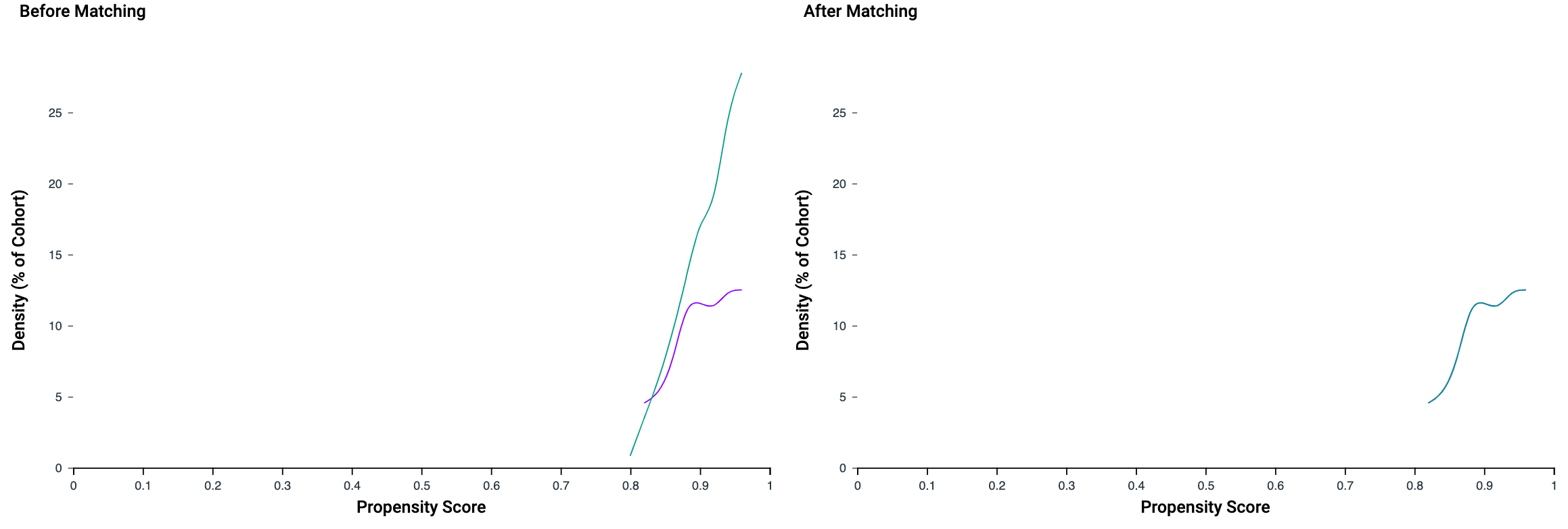

Supplement: Supplementary file 3 — Supplemental Files 5-8 [file 41392_2021_689_MOESM3_ESM.zip › Supplemental-files-H1+H2_(vent)/Propensity_Score_Density_Graph_Small.png]

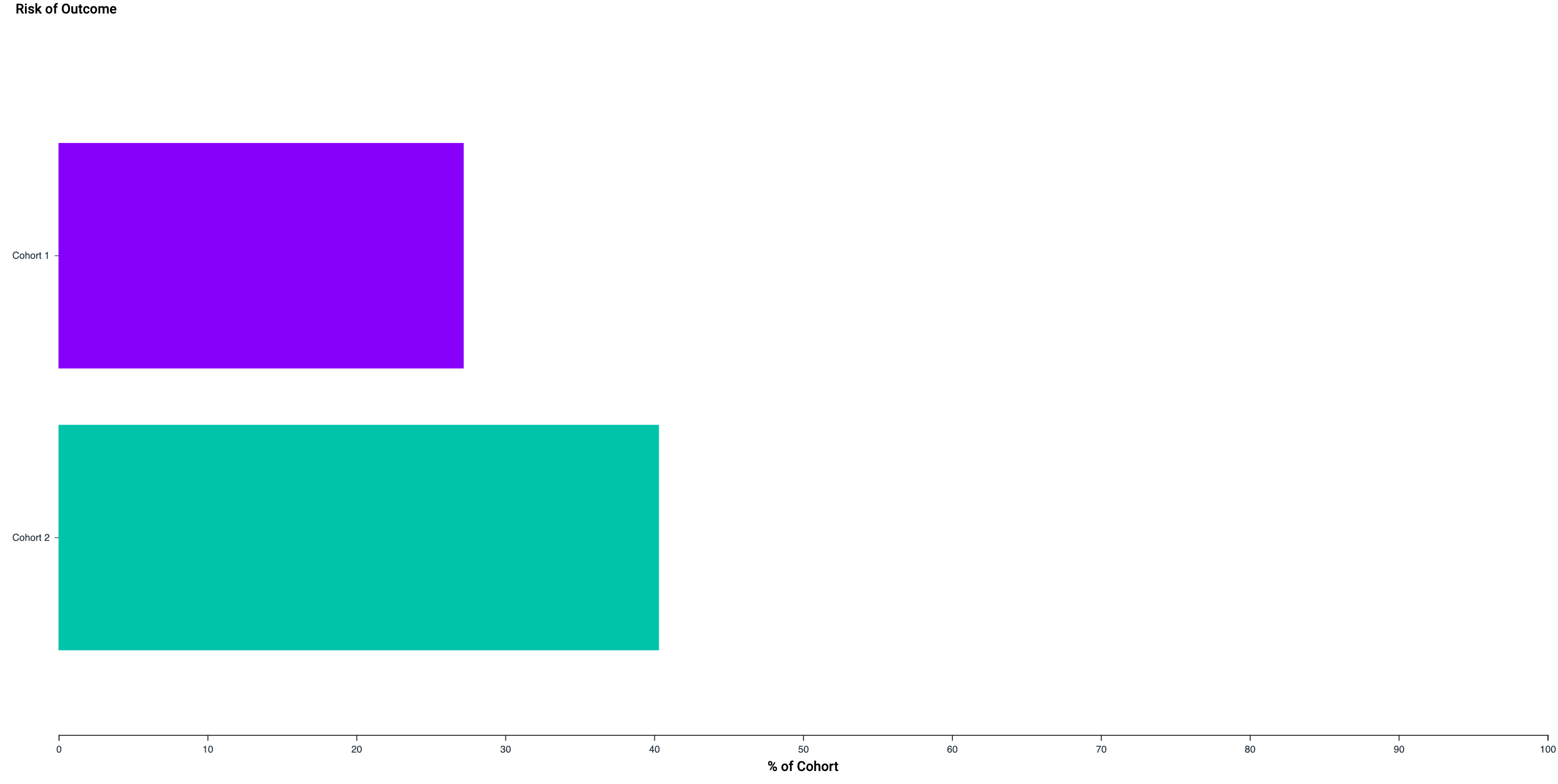

Supplement: Supplementary file 4 — Supplemental Files 9-12 [file 41392_2021_689_MOESM4_ESM.zip › Supplemental-files-ASA+famo_(vent)/Outcome_1_Result_a_MOA_graph_large.png]

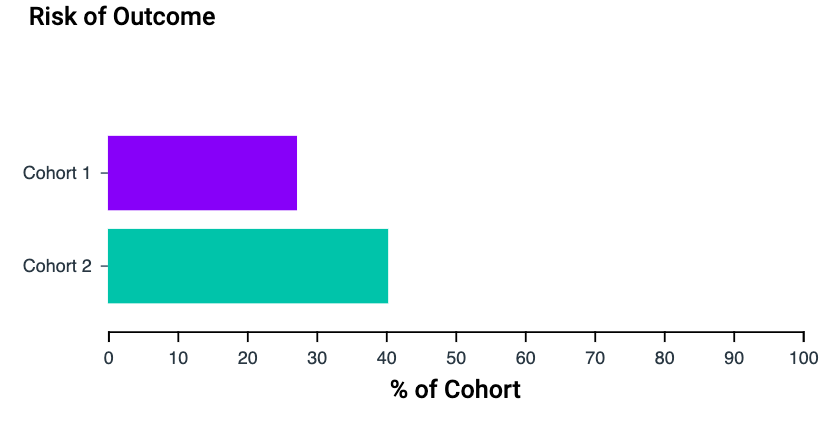

Supplement: Supplementary file 4 — Supplemental Files 9-12 [file 41392_2021_689_MOESM4_ESM.zip › Supplemental-files-ASA+famo_(vent)/Outcome_1_Result_a_MOA_graph_small.png]

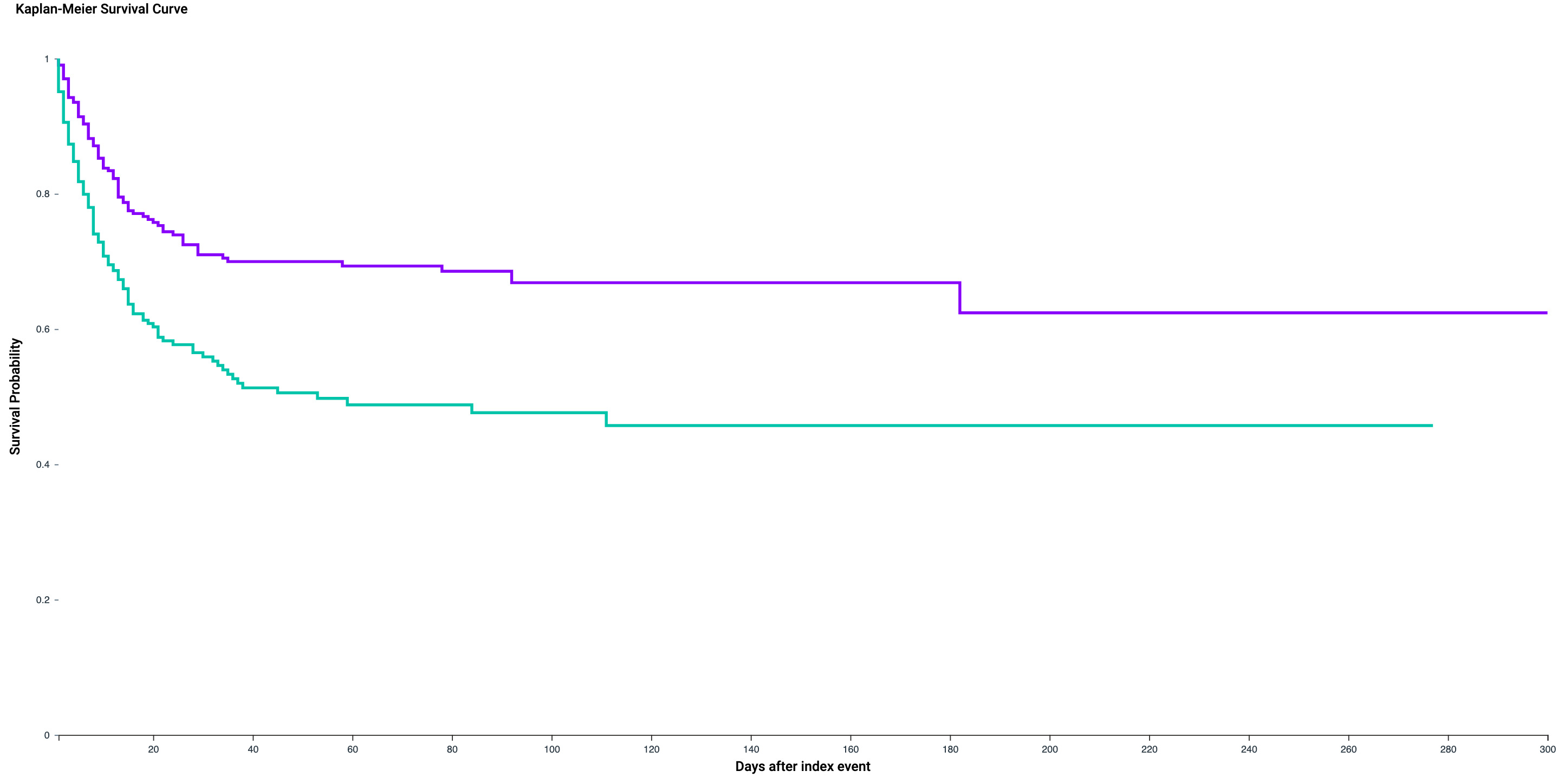

Supplement: Supplementary file 4 — Supplemental Files 9-12 [file 41392_2021_689_MOESM4_ESM.zip › Supplemental-files-ASA+famo_(vent)/Outcome_1_Result_b_KM_graph_large.png]

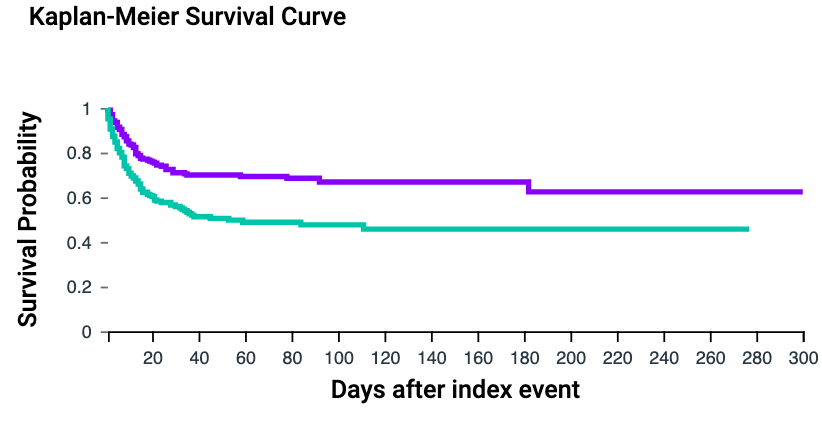

Supplement: Supplementary file 4 — Supplemental Files 9-12 [file 41392_2021_689_MOESM4_ESM.zip › Supplemental-files-ASA+famo_(vent)/Outcome_1_Result_b_KM_graph_small.png]

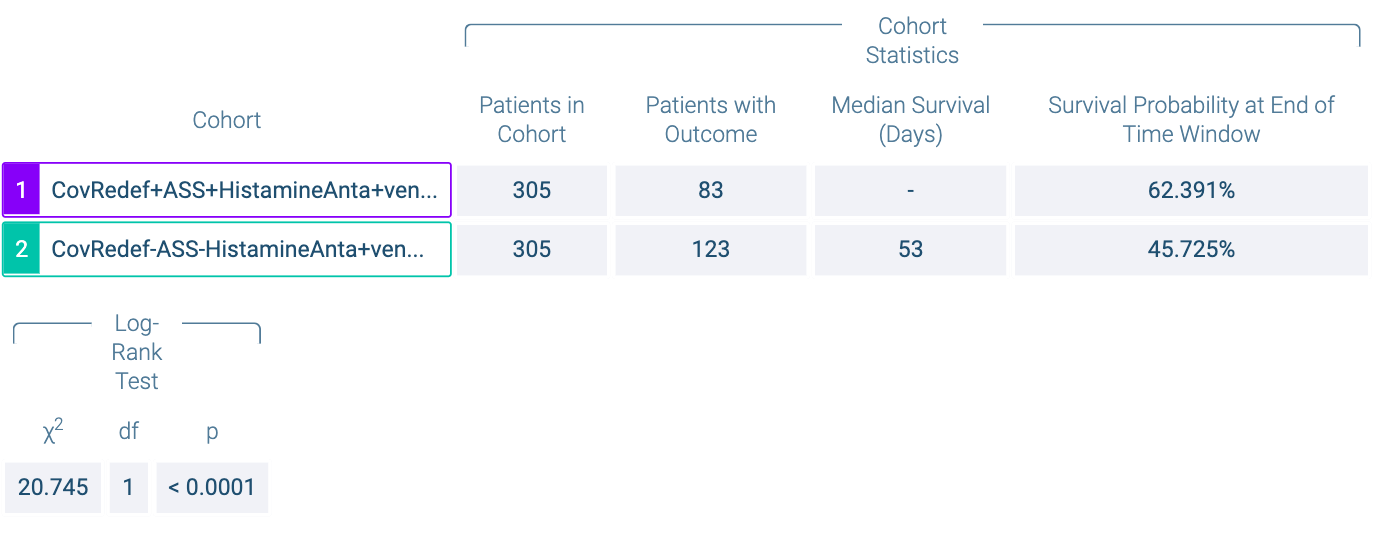

Supplement: Supplementary file 4 — Supplemental Files 9-12 [file 41392_2021_689_MOESM4_ESM.zip › Supplemental-files-ASA+famo_(vent)/Outcome_1_Result_b_KM_table.png]

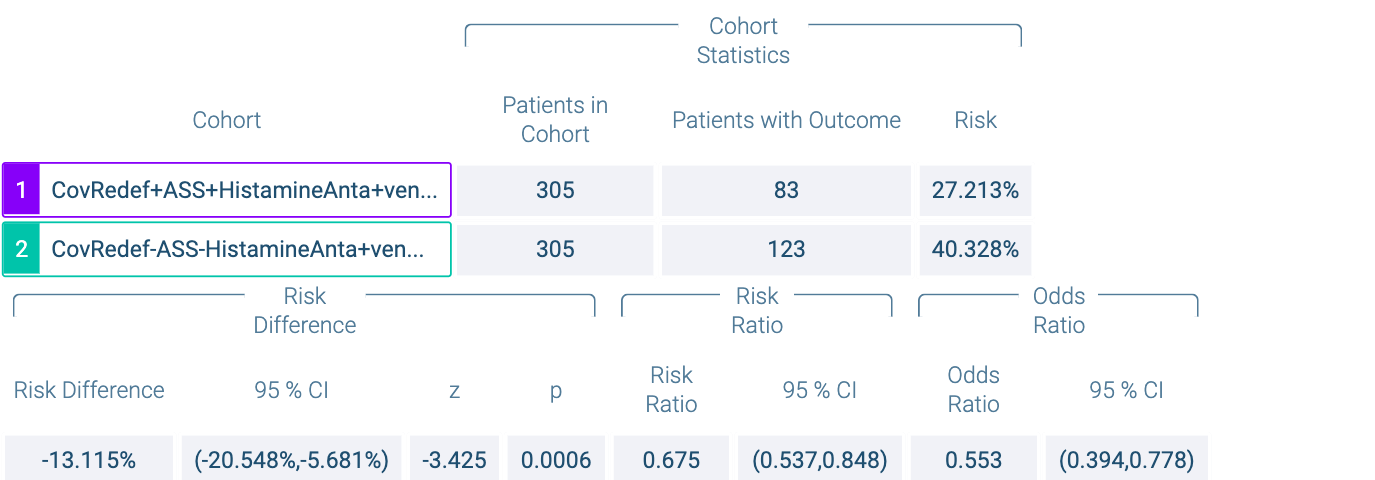

Supplement: Supplementary file 4 — Supplemental Files 9-12 [file 41392_2021_689_MOESM4_ESM.zip › Supplemental-files-ASA+famo_(vent)/Outcome_1_Result_a_MOA_table.png]

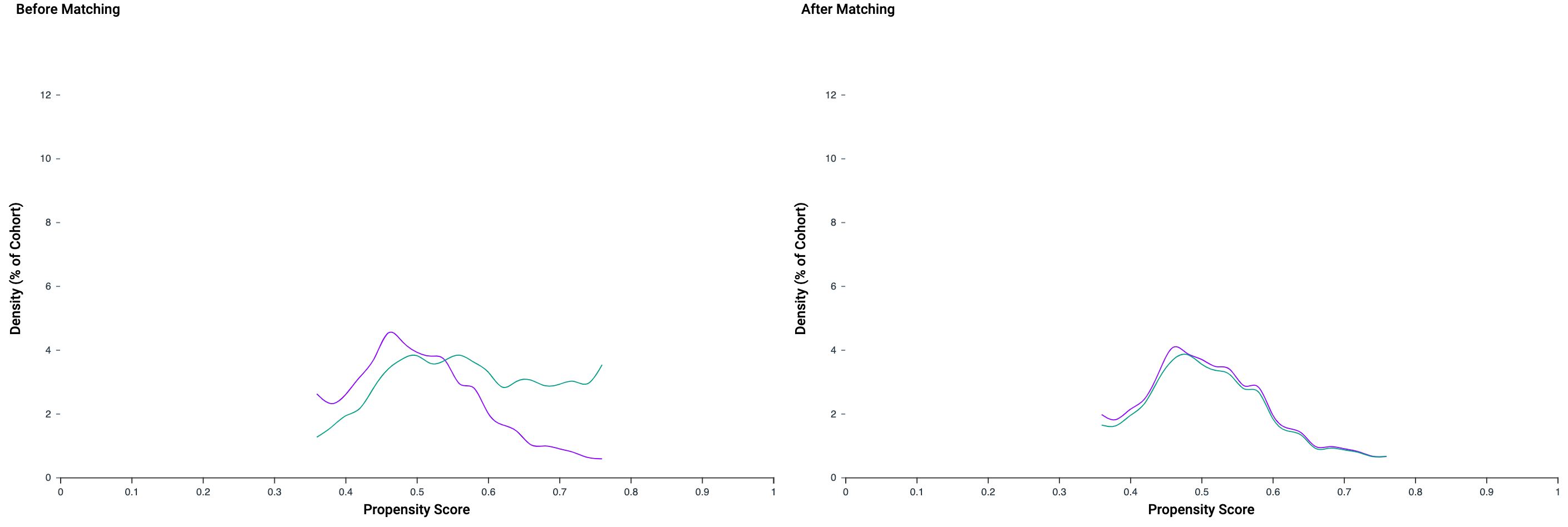

Supplement: Supplementary file 4 — Supplemental Files 9-12 [file 41392_2021_689_MOESM4_ESM.zip › Supplemental-files-ASA+famo_(vent)/Propensity_Score_Density_Graph_Large.png]

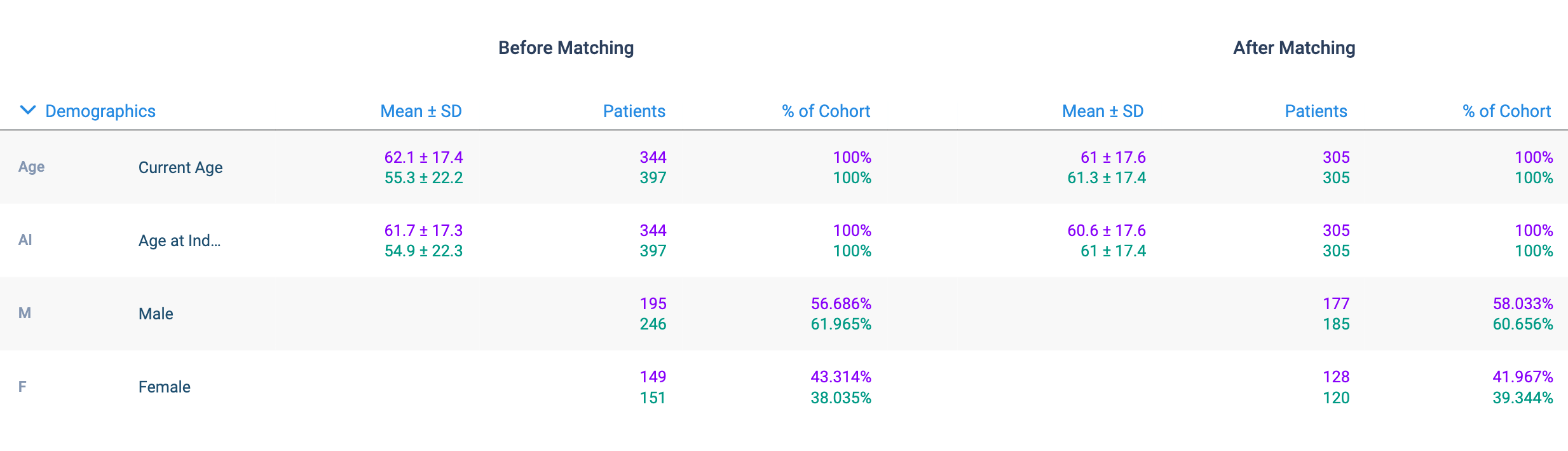

Supplement: Supplementary file 4 — Supplemental Files 9-12 [file 41392_2021_689_MOESM4_ESM.zip › Supplemental-files-ASA+famo_(vent)/Baseline_Patient_Characteristics.png]

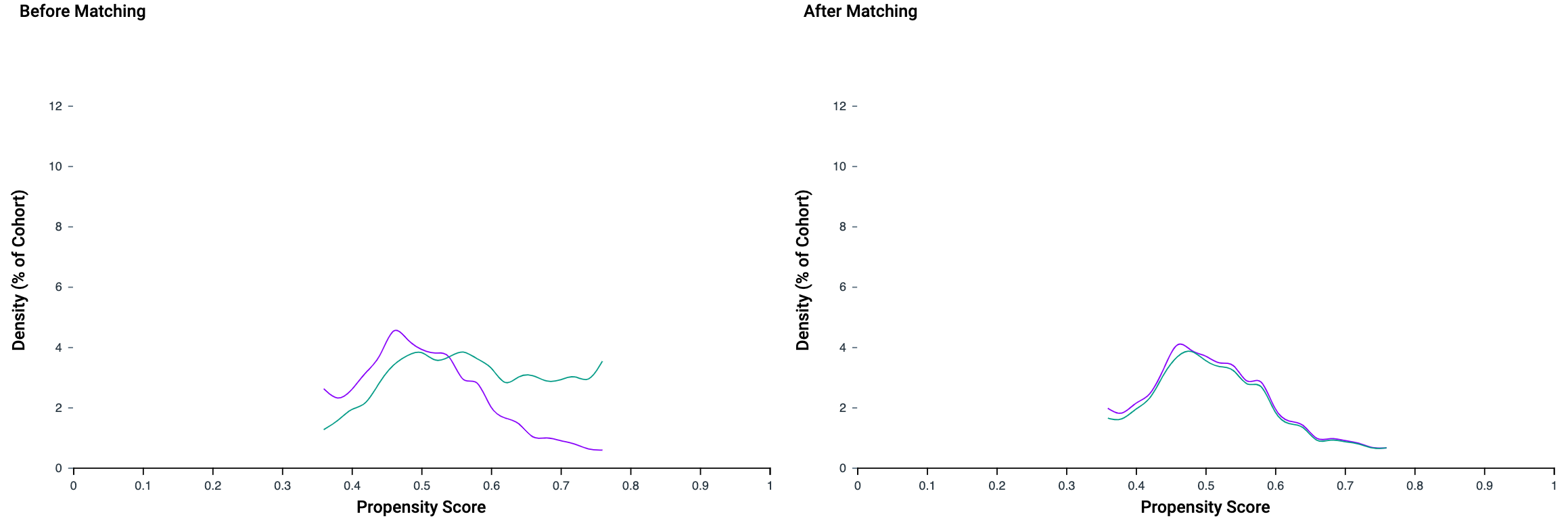

Supplement: Supplementary file 4 — Supplemental Files 9-12 [file 41392_2021_689_MOESM4_ESM.zip › Supplemental-files-ASA+famo_(vent)/Propensity_Score_Density_Graph_Small.png]
